# Quality of Sulfadoxine-Pyrimethamine Given as Antimalarial Prophylaxis in Pregnant Women in Selected Health Facilities in Central Region of Ghana

**DOI:** 10.1155/2016/9231946

**Published:** 2016-03-06

**Authors:** Danny F. Yeboah, Richmond Afoakwah, Ekene K. Nwaefuna, Orish Verner, Johnson N. Boampong

**Affiliations:** ^1^Department of Biomedical and Forensic Sciences, School of Allied Health Sciences, University of Cape Coast, Cape Coast, Ghana; ^2^Biotechnology and Nuclear Agriculture Research Institute, Ghana Atomic Energy Commission, Accra, Ghana; ^3^Department of Microbiology and Immunology, School of Medicine, University of Health and Allied Sciences, Ho, Ghana

## Abstract

The use of sulfadoxine-pyrimethamine (SP) as an intermittent preventive treatment (IPT) against malaria during pregnancy has become a policy in most sub-Sahara African countries and crucially depends on the efficacy of SP. This study sets out to evaluate the effectiveness of the SP given to the pregnant women in some selected health facilities in the Central Region of Ghana to prevent maternal malaria in pregnant women. A total of 543 pregnant women recruited from 7 selected health centres in Central Region of Ghana participated in the study. Parasite density of* Plasmodium falciparum* was determined from peripheral blood of the pregnant women using microscopy. High performance liquid chromatography (HPLC) and dissolution tester were used to determine the quality of the SP. Malaria infection was recorded in 11.2% of pregnant women who had a history of SP consumption. SP failed the dissolution test. Pregnant women who did not receive IPT-SP were 44%. Low haemoglobin level was recorded in 73.5% of the pregnant women. The results indicated that SP was substandard. IPT-SP is ineffective in preventing malaria infection.

## 1. Introduction

Over 50 million pregnant women a year are exposed to malaria resulting in 2 500–10 000 maternal deaths annually [1], with at least 60 percent of them in Africa. In sub-Sahara Africa, 25 million pregnant women are at risk of* Plasmodium falciparum* infection every year, and one in four women has evidence of placental infection at the time of delivery [2]. In high transmission areas, malaria is associated with maternal anaemia, potentially responsible for maternal death when severe, and low birth weight due to both prematurity and intrauterine growth retardation [3]. World Health Organization (WHO) policy for malaria prevention and control during pregnancy in areas of stable transmission emphasizes a preventive package of intermittent preventive treatment (IPT), insecticide-treated bed nets (ITNs), and effective case management of malaria illness and anemia.

The 2014 WHO policy update for intermittent preventive treatment in pregnancy (IPTp) recommends that doses should be delivered at each antenatal care visit after the first trimester, with a minimum of three doses received during each pregnancy [4]. The most effective drug for IPT is sulfadoxine-pyrimethamine (SP) because of its safety for use during pregnancy, effectiveness in reproductive-age women, and feasibility for use in programmes, as it can be delivered as a single-dose treatment under observation by the health worker [5]. In areas of IPTp with SP implementation, increasing resistance to SP is a growing challenge. Already in parts of Africa and Southeast Asia, the effectiveness of SP in IPTp is being threatened by increasing levels of resistance to SP [6].

Following the implementation of IPTp-SP, clinical and parasitological parameters of pregnant women attending ANC at Agogo Hospital in Ghana improved [7]. This improvement was, however, short-lived due to resistance to SP [8]. The usefulness of SP for IPT in countries facing moderate to high levels of SP resistance needs to be evaluated [5]. SP treatment failure occurred in pregnant women in central Ghana during 2003-2004 [9]. Treatment failure might be a result of the administration of substandard standard drugs or the development of resistance of the parasite to the drug. The purpose of this study is to determine whether IPT with SP is able to eliminate malaria parasites from the peripheral blood of pregnant women.

## 2. Methods

### 2.1. Study Area and Population

**Figure 1:**
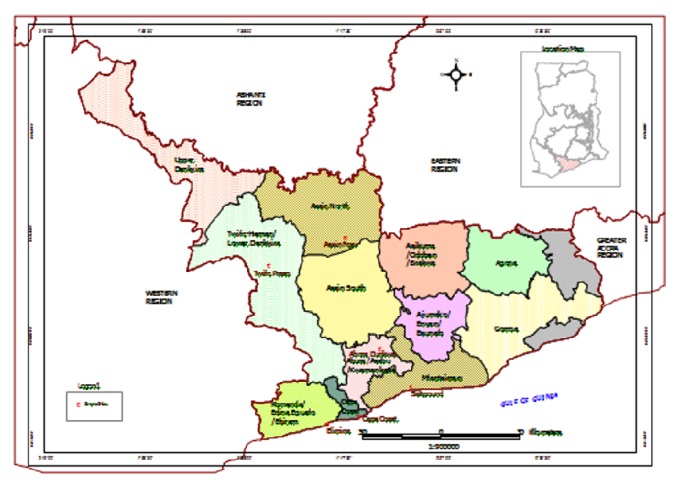
Map of Central Region of Ghana showing districts.

The study was carried out in 7 health centres in 6 selected towns in the Central Region of Ghana. The Central Region is located at the southern part of Ghana. It is bordered by the Ashanti and Eastern regions to the north, Western region to the west, Greater Accra region to the east, and the Atlantic Ocean to the south. The Central Region is situated within latitudes 6°15N and 5°N and longitudes 2°15W and 45°E. The region is partitioned into 13 districts (Figure 1). The region has an area of about 9.826 km^2^ [10] and a population of about 1.6 million [11]. Central Region is made up of two ecological zones, the coastal and the forest zones. Towns from which subjects were selected are Assin Foso, Twifo Praso, and Abura Dunkwa located in the forest zone and Cape Coast, Saltpond, and Elmina in the coastal zone. The populations of the towns are as follows: Cape Coast, 82,292; Saltpond, 16,212; Elmina, 21,103; Assin Foso, 22,837; Twifo Praso, 9,011; Abura Dunkwa, 8,439. Fishing, farming, and petty trading are the main occupation of the inhabitants. Two rainy seasons occur in the Central Region. The main wet season is from April to September and the dry season is from November to March (http://www.travel-to-discover-ghana.com/). The study was conducted in the two seasons: March in the dry season and August in the rainy season in the year 2009.

The study was carried out on pregnant women who had reported their routine monthly antenatal visit at the antenatal clinics of seven randomly selected health facilities in Central Region of Ghana. The selected facilities included Cape Coast Metropolitan Hospital, Ewim Urban Health Centre at Cape Coast, St. Francis Xavier Hospital at Assin Foso, Twifo Praso Government Hospital, Elmina Urban Health Centre, Saltpond District Hospital, and Abura Dunkwa District Hospital.

### 2.2. Ethical Consideration

Ethical approval was obtained from the University of Cape Coast Institutional Review Committee. Approval was also sought from the Central Regional Directorate of the Ghana Health Service before commencement of this research. The rationale of the study was explained to the subjects with the assistance of the midwives. Voluntary participation was emphasized and ensured. Participant anonymity was also ensured. Written informed consent from each consenting pregnant woman was sought before sample collection.

### 2.3. Participant Recruitment and Sample Collection

Participants were randomly recruited from pregnant women who had reported their routine monthly antenatal visit at the antenatal clinics of seven randomly selected health facilities in Central Region of Ghana. Blood was collected from participants upon their next antenatal visit from the day of recruitment. This was to ensure that blood collection was done at least a week after the consumption of SP by the pregnant women. The number of SP doses each participant has consumed was recorded. Participants were, thus, categorized as no-SP dose, one-SP dose, two-SP dose, and three-SP dose groups depending on the number of SP doses they had consumed at the point of sample collection.

Five millilitres (5 mL) of blood sample was collected from each participant into tubes containing EDTA by trained and licensed medical laboratory technologists in the various health facilities. All blood samples collected were stored on ice and transported to the Parasitology Research Laboratory of the Department of Biomedical and Forensic Sciences, University of Cape Coast

### 2.4. Estimation of Parasite Density

Giemsa-stained thick blood films were prepared and the slides were observed under the light microscope using ×100 objective lens [12]. Parasite density was determined by counting the number of malaria parasites against 200 white blood cells (WBCs). A standard WBC count of 8000 per microlitre (*μ*L) of blood is assumed for each participant. In cases where less than ten parasites were counted against 200 WBCs, counting of parasites continued until 500 WBCs were identified. A slide was considered negative if no parasite was found after counting 500 WBCs [13]. Parasite density was categorized as follows: 0 (aparasitaemic), 1–999, 1000–9999, and ≥10,000 [14]. Such a classification is appropriate as higher parasite densities correspond to greater severity of the infection and acute phase of malaria [15].

### 2.5. Determination of Haemoglobin

The haemoglobin level of each sample collected was estimated using a Cell Dyn 1800 automated blood analyzer. A participant was considered anaemic if his/her haemoglobin level was below 11 g/dL.

### 2.6. Drug Sourcing

Three SP drugs were sampled for this study between October and November 2009. For the purpose of confidentiality, the original names and batch numbers of the drugs have been withheld. The drugs were coded as Drugs I, II, and III. Drug I is locally manufactured and given to pregnant women at the antenatal care clinics as prophylaxis. Drug II is also locally manufactured and sold on the open market in pharmacies and licensed chemical shops. Drug III is an imported product commonly found in pharmacies and licensed chemical shops. Drug I was obtained from antenatal clinics in Cape Coast, Saltpond, Elmina, Twifo Praso, Assin Foso, and Abura Dunkwa, while Drugs II and III were obtained from pharmacies and licensed chemical shops in these same areas through mystery buying. All three drugs sampled for this study had more than a year to expire. The manufacturing and expiry dates for the Drugs I were September 2007–September 2011 and those for both Drugs II and III were April 2008–April 2012. The health facilities and retail shops stored the drugs in well ventilated rooms at room temperature and kept them away from sunlight. After sampling, the drugs were stored in their various packages at room temperature until drug analysis was carried out at the laboratory of Ghana Standard Authority in February 2010.

### 2.7. Drug Analyses

In assessing the quality of the SP, the chemical or biological assay and the dissolution methods were employed. Reference standard solution of SP was prepared by dissolving approximately 0.5 g (500 mg) of sulfadoxine and 0.025 g (25 mg) of pyrimethamine in 10 mL methanol and diluted with mobile phase to 100 mL. Five replicate injections of a system suitability preparation (standard solution) were chromatographed and the peak areas were recorded [16].

Twenty tablets of each brand of SP drug were weighed and the average weight was determined. The 20 tablets of each brand were powdered separately with mortar and pestle. An accurately weighed amount of the finely ground powder, equivalent to 0.5 g sulfadoxine and 0.025 g pyrimethamine of each sample, was dissolved in 10 mL of methanol with the aid of an ultrasonic bath. It was then diluted with mobile phase to 100 mL, mixed, and filtered [16]. The samples were then introduced into an HPLC. Three replicates of each sample were run and the average peak area was recorded. The weight in milligrams of sulfadoxine and pyrimethamine in a tablet of each brand was determined. Using these results, the amounts of sulfadoxine and pyrimethamine per tablet were calculated and expressed as percentage of the label claim [17].

Tablet dissolution was performed in the ERWEKA DT 600 dissolution apparatus using 6 tablets of each product. Dissolution of the antimalarial products was carried out using 1 litre of 0.01 M pH phosphate buffer solution (sodium hydroxide and potassium dihydrogen orthophosphate, Fisher Scientific) and heated to a temperature of 37°C, with a rotor speed of 75 revolutions per minute (rpm). The 6 tablets were introduced into the dissolution vessels and dissolution was carried for 30 minutes [18]. Samples (2 mL) of the dissolution media in the different vessels were withdrawn after 30 minutes and were transferred into HPLC reaction vial and then introduced into the HPLC machine for analysis for sulfadoxine and pyrimethamine content according to the HPLC method described in the USP for dissolution testing of these tablets [19].

### 2.8. Data Analysis

The data collected were recorded into a precoded case record forms. Thereafter, the data were transferred to Statistical Package for the Social Sciences (SPSS) version 10 for analysis. Descriptive statistics such as means and standard deviations were used to summarize quantitative variables, while categorical variables were summarized with proportions. The student *t*-test was used to compare two mean values while the one-way analysis of variance (ANOVA) was used to compare mean values in more than two groups. The Chi-square test was used to investigate associations between categorical values and also to analyze differences in proportions. For significant associations, 95% confidence intervals were computed. A *P* value of less than 0.05 was considered statistically significant.

## 3. Results

A total of 543 pregnant women were recruited into the programme. The ages of the participants ranged from 16 to 44 of which the majority 444 (82%) of the women were between the ages of 18 and 34 years. The pregnancies were from 5 to 9 months. The women who were pregnant for the first time constituted 32% (172), whilst 68% (371) of them have had between 1 and 9 pregnancies. About 77% of the subjects were employed.

Malaria parasites were found in the peripheral blood of pregnant women who had taken SP and those who had not taken. Of the 543 pregnant women, 304 (56.0%) had taken Drug I (SP). Those who had not taken SP were 239 (44.0%). A total of 63 pregnant women out of the 543 (11.6%) had malaria parasites in their blood. Out of the 63 infected pregnant women, 34 (54.0%) had taken SP and 29 (46.0%) had not taken SP (Table 1). There was no significant difference between those who had taken SP and were yet infected and those who had not taken SP and were infected (*χ* ^2^ = 0.27; *P* value = 0.871).

The haemoglobin levels of the pregnant women were generally low. As many as 399 (73.5%) of the pregnant women had low haemoglobin, whilst 144 (26.5%) had normal haemoglobin. Forty-nine pregnant women representing 77.8% of the infected pregnant women had low haemoglobin (below 11 g/dL), whilst 72.9% of the uninfected pregnant women also had low haemoglobin (Table 1). There was no significant difference in the haemoglobin levels of the pregnant women who had malaria parasites in their blood and those who had no malaria parasites in their blood (*χ* ^2^ = 0.351; *P* value = 0.553).

There was relatively greater parasite density in the multigravidae than in the primigravidae. There were 33.9% (184) and 66.1% (359) primigravidae and multigravidae, respectively. The infected primigravidae and multigravidae were 25 (39.7%) and 38 (60.3%), respectively. Forty-five infected pregnant women had parasite densities within 1–999 parasites/*μ*L. Out of this number, 16 (35.6%) were primigravidae compared to 29 (64.4%) who were multigravidae. Eighteen of the infected pregnant women had parasite densities between 1000 and 9999 parasites/*μ*L and out of this number 9 (50.0%) were primigravidae, while the remaining 9 (50.0%) were multigravidae (Table 2). The difference in the parasite densities between primigravidae and multigravidae was not significant (*χ* ^2^ = 256.0; *P* value = 1.000).

### 3.1. SP Drug Test

Drug I and Drug III passed the drug assay test in that the average percentage composition of both sulfadoxine and pyrimethamine in these drugs fell within the 90.0%–110.0% range. Drug II failed the assay test because the average percent composition of pyrimethamine in it was below 90.0 percent (Table 3). Drug dissolution test was not performed for Drug II, because it failed the first test. Drug III passed the drug dissolution test. The percentage of both sulfadoxine and pyrimethamine of Drug III dissolved in the phosphate buffer in each of the vessels was not less than 60.0 percent. Drug I failed the drug dissolution test. The percentage of pyrimethamine of Drug I dissolved in the phosphate buffer in each of the vessels was less than 60.0 percent (Table 4). Moreover, the percentage of sulfadoxine of Drug I dissolved in the phosphate buffer in two of the vessels was less than 60.0 percent.

## 4. Discussion

All participants were eligible to take SP; however, only 56% had used IPTp-SP. This rather low compliance to IPTp-SP has been explained to be due to late first ANC clinic enrolment [20, 21]. Irrespective of the low compliance to IPTp-SP,* Plasmodium* infection was comparable between participants who had consumed SP and those who had not (*P* = 0.553), suggesting the ineffectiveness of Drug I, which is used in IPTp-SP in the country. Drug failure may be due to either development of parasite resistance to the drug or inadequate drug levels, through suboptimal dosing, poor quality of the antimalarial, poor absorption, or poor metabolism to the active metabolites [22]. Drug assay and the dissolution test conducted on Drug I showed poor dissolution of the drug. Thus, the bioavailability of the drug may be compromised, leading to its ineffectiveness. This will have an implication in the spread of SP-resistant parasite strains.

Generally, the pregnant women were anaemic. Anaemia is one of the most important consequences of malaria infection during pregnancy. Anaemia during pregnancy can cause low birth weight and low birth haemoglobin in the infant. This consequently may lead to increased morbidity for the infant [23]. Relatively, there was no significant difference between the haemoglobin levels of pregnant women who had malaria infection and those who did not. This shows that irrespective of malaria, the haemoglobin levels of the pregnant women were generally low. This confirms the multifactorial etiology of anaemia in pregnancy including iron deficiency, folate deficiency, poor diet, hookworm infections, and malaria [24]. Two other known factors which contribute to development of iron deficiency anaemia in pregnancy are first the woman's iron stores at the time of conception and second the amount of iron absorbed during gestation [25]. There is a normal reduction in haemoglobin level at the beginning of pregnancy followed by a slight rise towards the end of pregnancy [26]. The initial reduction has been explained to result from increased red cell mass and demands of the foetus which exceed iron intake with consequent reduction in iron stores of the woman's body [26]. This is why iron supplementation in pregnancy has become a standard and routine practice as a preventive treatment for iron deficiency anaemia in pregnancy in developing countries [27].

In Ghana, the government provides free antenatal care. Pregnant women are given malaria prophylaxis in the form of SP tablets (Drug I) obtained from a particular pharmaceutical firm. SP from other sources are sold in pharmaceutical shops. Drug I and Drug III passed the drug assay test, implying that they contain the right quantities of the active ingredients, sulfadoxine and pyrimethamine. However, only Drug III passed the drug dissolution test. The test for dissolution determines the amount of active ingredient that is released and available for absorption. Poor manufacturing practices, poor storage of a product, and the use of incorrect excipients will lead to poor dissolution profiles [18]. The failure of Drug I to pass the drug dissolution test is an indication that a lesser amount of the drug is able to dissolve within the specified period and thus results in poor availability for absorption, rendering the drug ineffective. This situation exposes the pregnant women to the risk of malaria infection thereby defeating the aim of IPTp in Ghana. This may account for the observed persistent malaria infection in pregnant women who have taken SP (Drug I), confirming the hypothesis that SP is incapable of preventing malaria in the pregnant women.

## 5. Conclusion

Drug I, being used in IPTp-SP in Ghana, is of low quality and thus may be ineffective in prevention and treatment of malaria in pregnancy.

## Figures and Tables

**Table 1:** IPTp-SP compliance and haemoglobin level against malaria in pregnant women.

Category	Malaria status		*P* value
	Infected	Noninfected	
	*n* (%)	*n* (%)	
IPTp-SP compliance			*0.871*
Yes	34 (54.0%)	270 (56.3%)	
No	29 (46.0%)	210 (43.7%)	
Total	63 (100%)	480 (100%)	
Haemoglobin level			*0.553*
Low	49 (77.8%)	350 (72.9%)	
Normal	14 (22.2%)	130 (27.1%)	
	63 (100%)	480 (100%)	

*P* value based on Chi-square test.

**Table 2:** Comparison of malaria parasite density between primigravidae and multigravidae.

Category	Gravidity		*P* value
	Primigravida	Multigravida	
	*n* (%)	*n* (%)	
Malaria status			*1.000*
Infected	25 (13.6%)	38 (10.6%)	
Noninfected	159 (86.4%)	321 (89.4%)	
Total	184 (100%)	359 (100%)	
Parasite density			*1.000*
0	159 (86.4%)	321 (89.4%)	
1–999	16 (8.7%)	29 (8.1%)	
1000–9999	9 (4.9%)	9 (2.5%)	
≥10000	0 (0%)	0 (0%)	
Total	184 (100%)	359 (100%)	

*P* value based on Chi-square test.

**Table 3:** Calculated weight in milligram and percentage composition of sulfadoxine and pyrimethamine in Drugs I, II, and III.

	Weight of sulfadoxine	Percentage composition	Weight of pyrimethamine	Percentage composition
Drug I	Per tablet (mg)	(%)	Per tablet (mg)	(%)
1	485.72	97.14	21.88	87.52
2	490.36	98.07	23.92	95.68
3	489.52	97.90	23.78	95.12
Average		97.71		92.76
Drug II				
1	485.31	97.06	18.39	73.56
2	485.88	97.18	18.89	75.56
3	485.62	97.90	19.16	76.64
Average		97.12		75.24
Drug III				
1	497.76	99.55	22.51	90.04
2	498.00	99.60	23.72	94.80
3	498.76	99.75	22.85	91.40
Average		99.63		92.12

(mg): milligram; (%): percentage.

**Table 4:** Recorded peak responses of five replicate injections and calculated percentage dissolution of sulfadoxine and pyrimethamine in Drugs I and III.

	Average peak area of sulfadoxine (mV)	Percentage of sulfadoxine dissolved (%)	Average peak area of pyrimethamine (mV)	Percentage of pyrimethamine dissolved (%)
Drug I				
1	6892774	59.42	90182	20.20
2	7076779	61.00	114852	25.74
3	6991092	60.27	90667	20.31
4	7017232	60.49	105682	23.67
5	6981546	60.18	110563	24.76
6	6889575	59.39	115472	25.86
Drug III				
1	10898130	93.94	358382.5	80.27
2	10516241	90.65	347357	77.80
3	10790608	93.02	305035	68.32
4	10811924	93.20	314093.5	70.35
5	10679338	92.06	298327.6	66.82
6	10592611	91.31	306223.3	68.59

(mV): millivolts.
